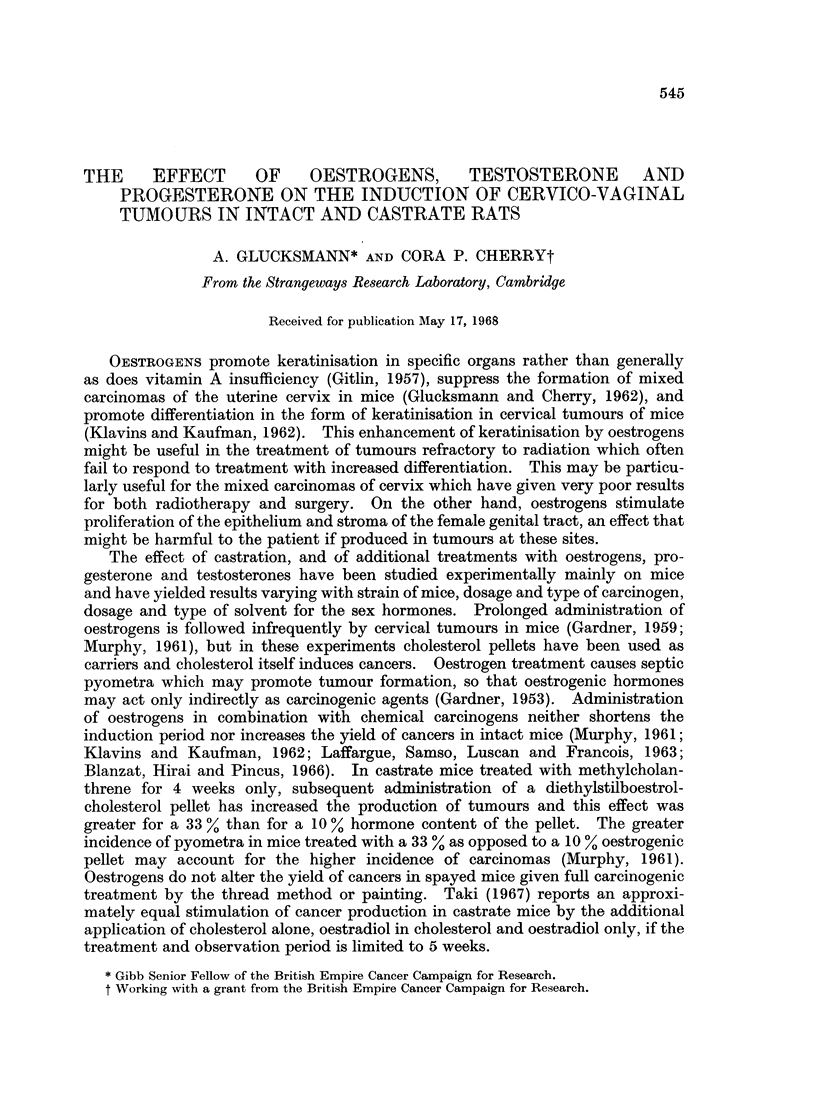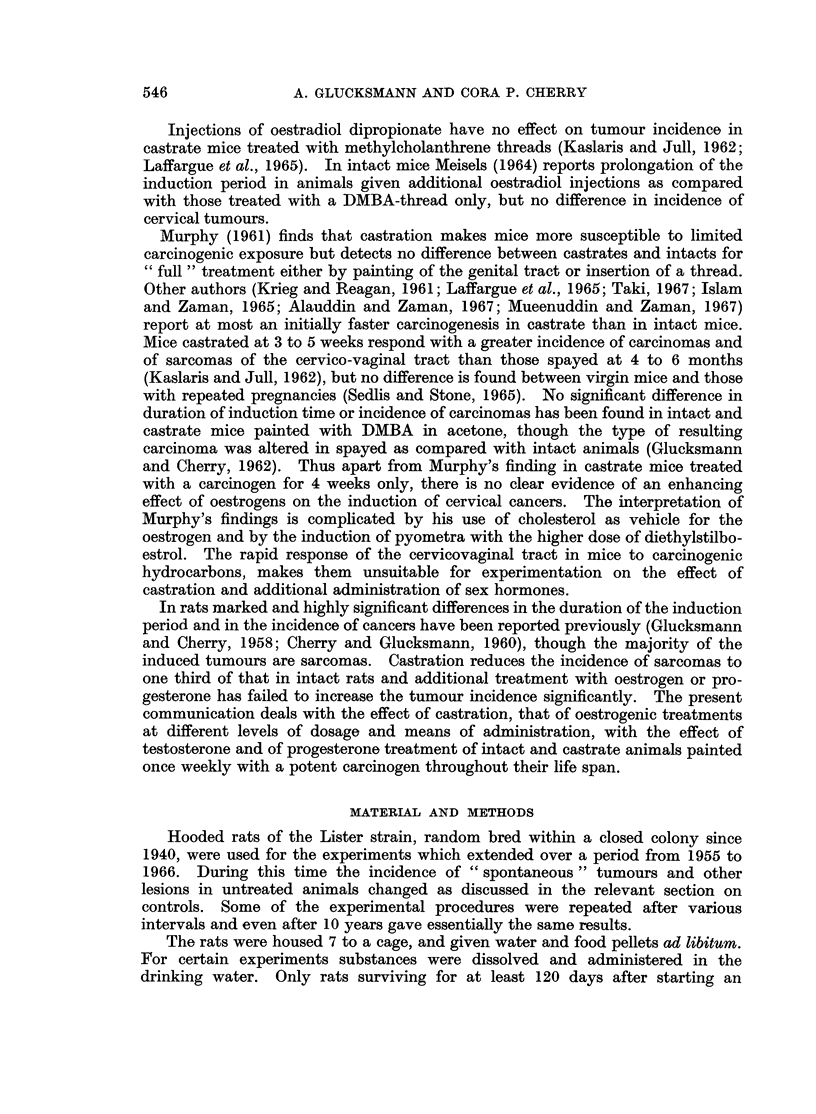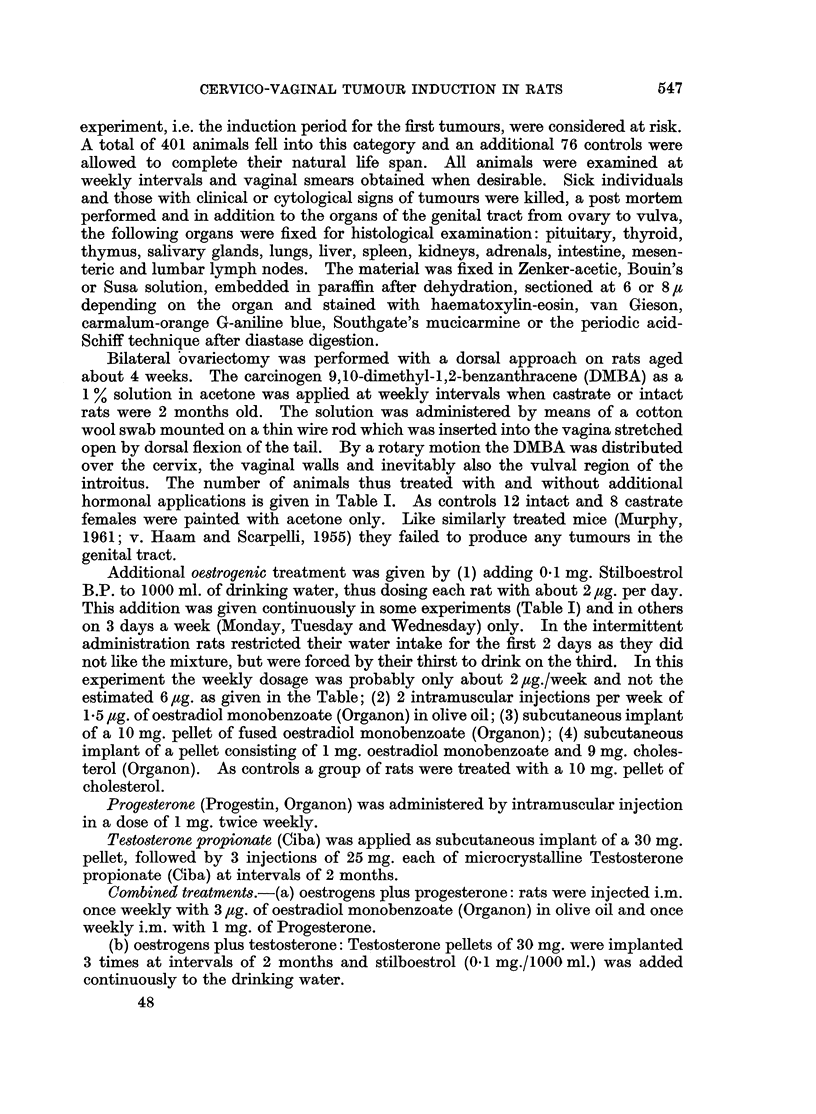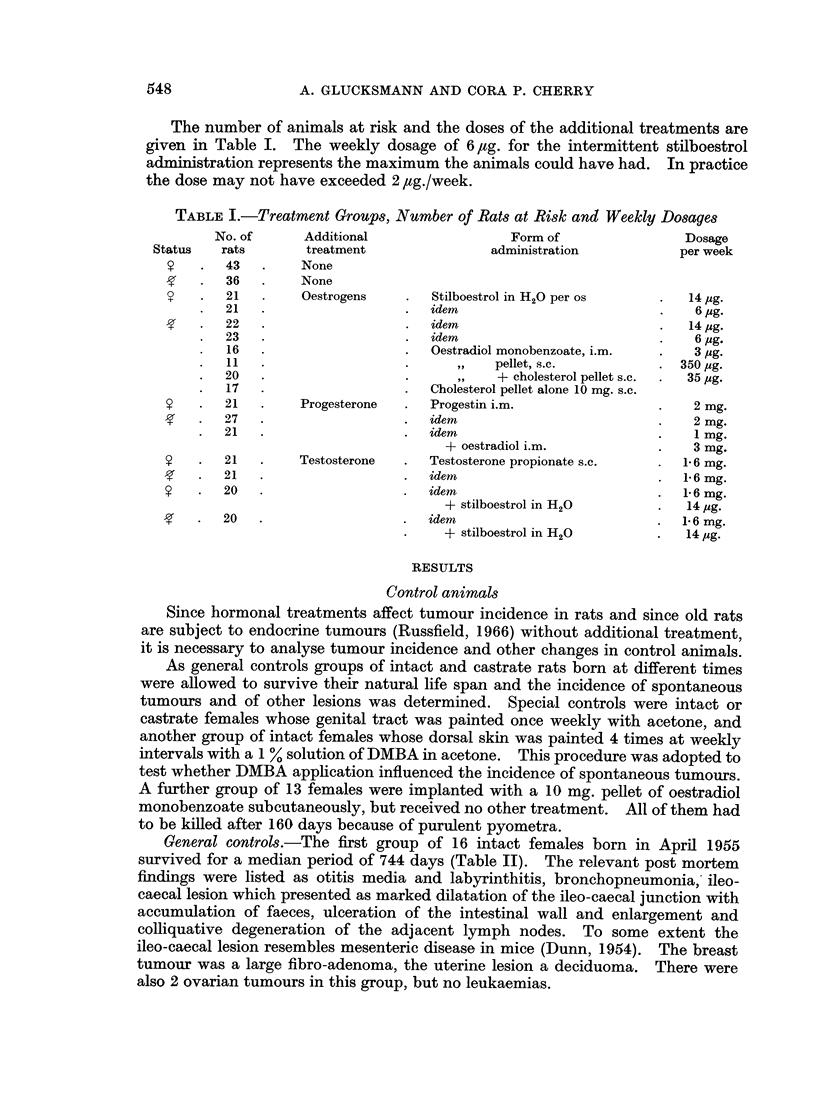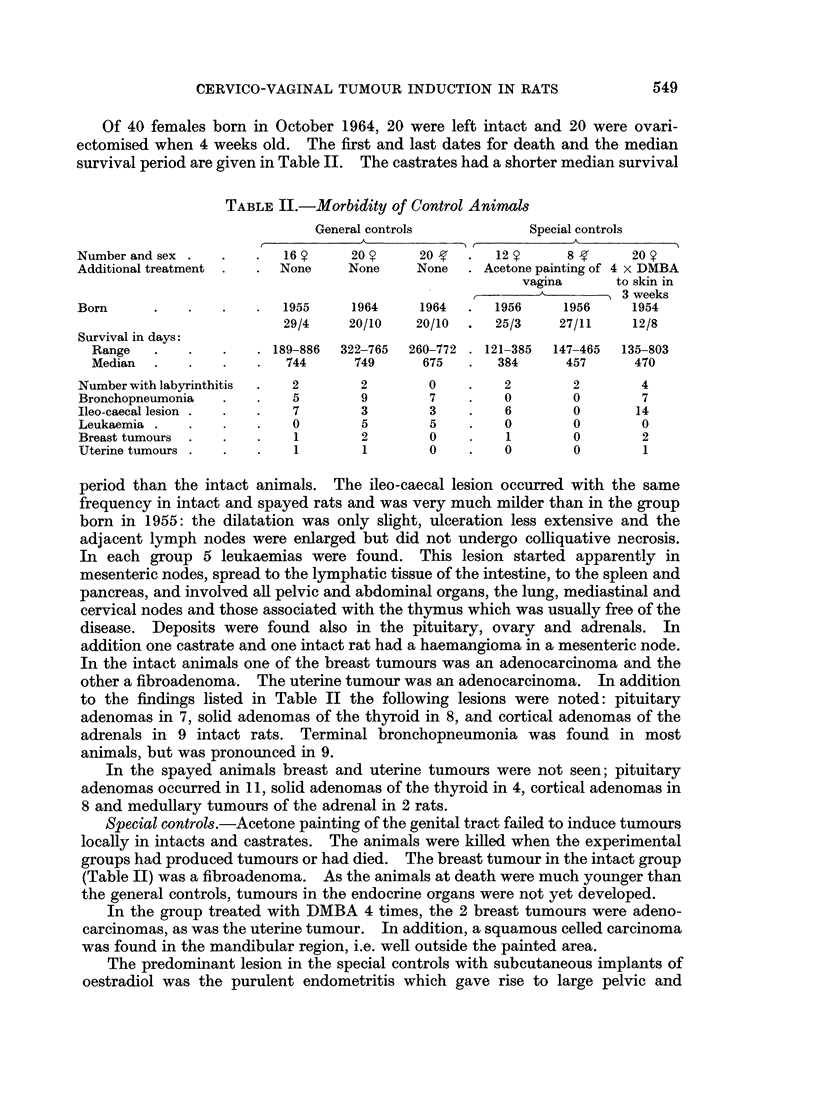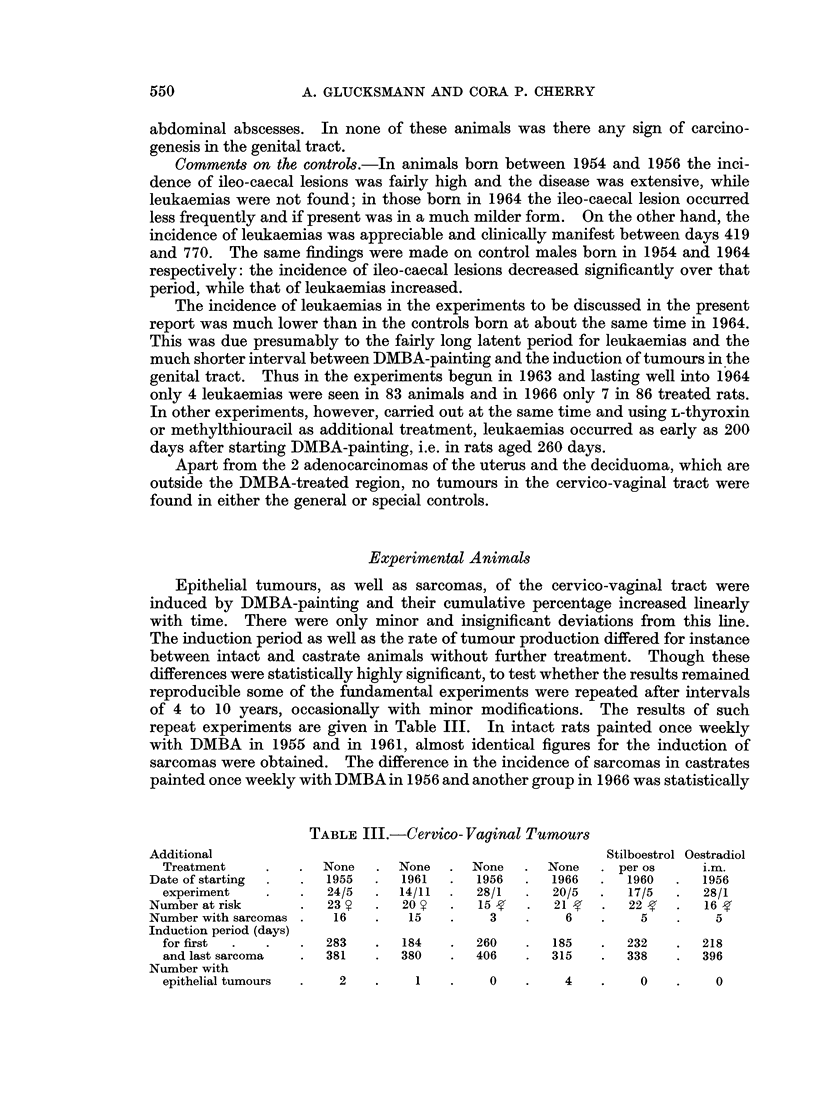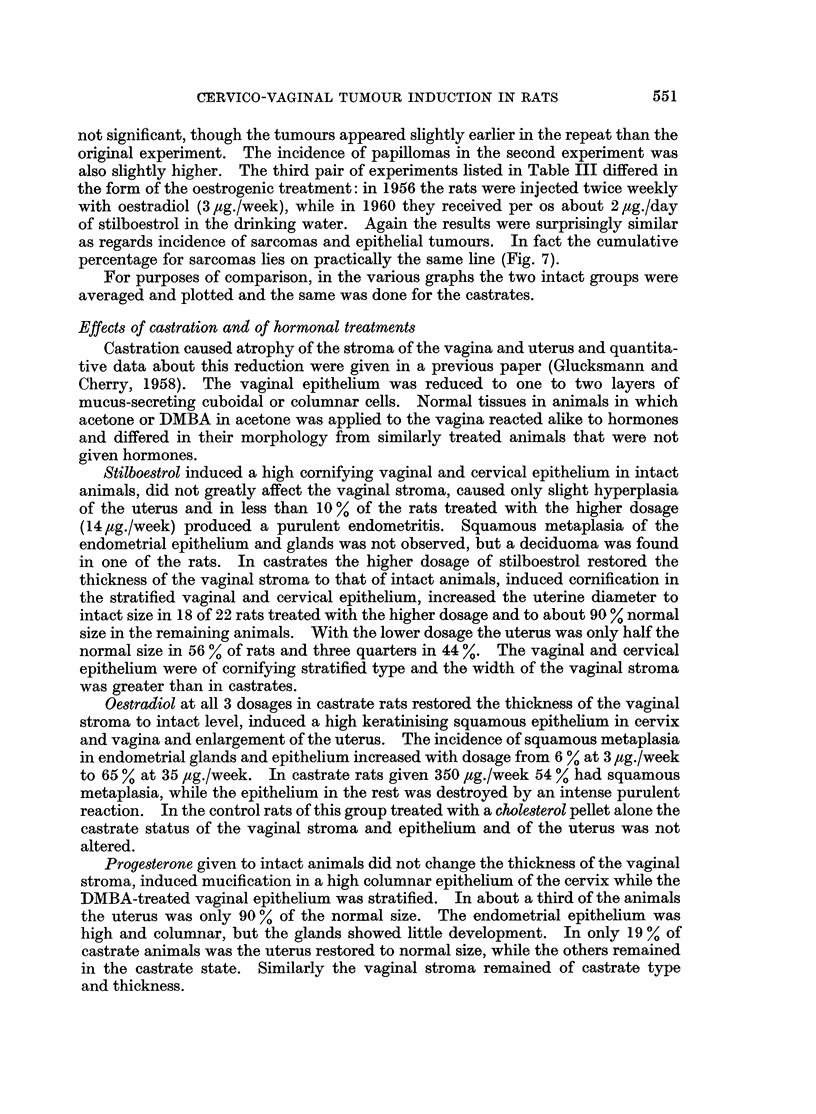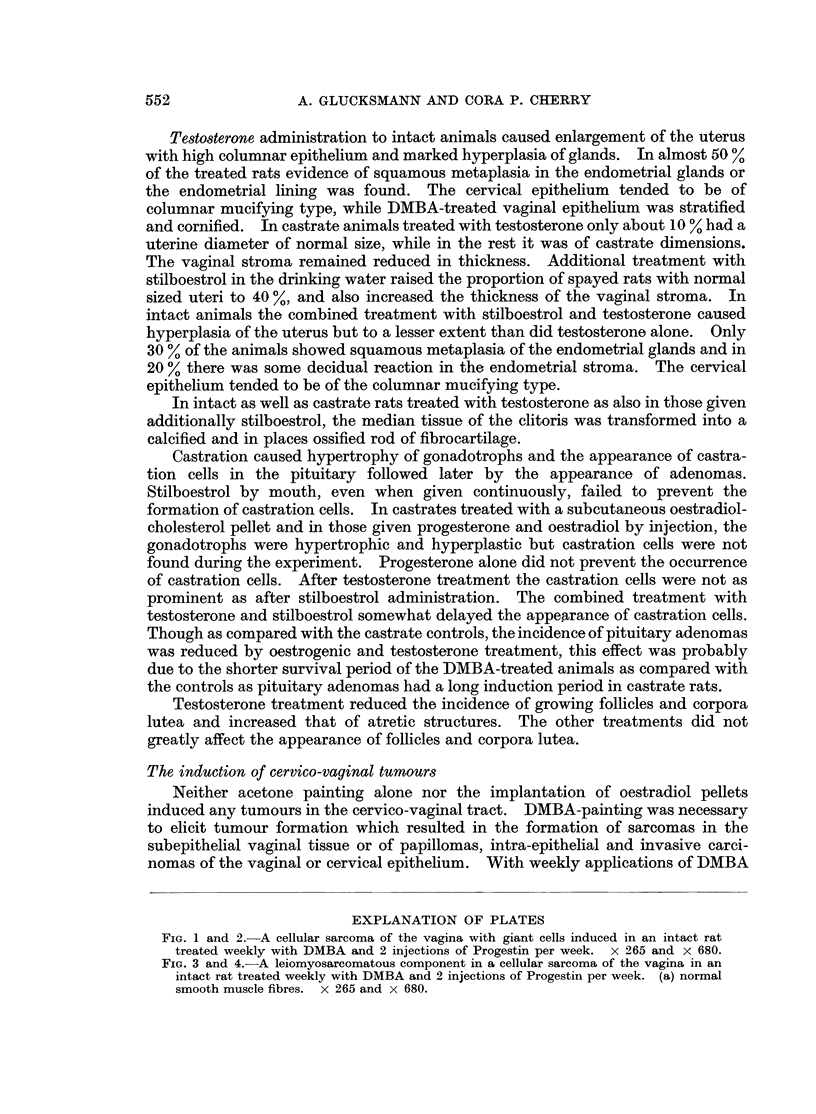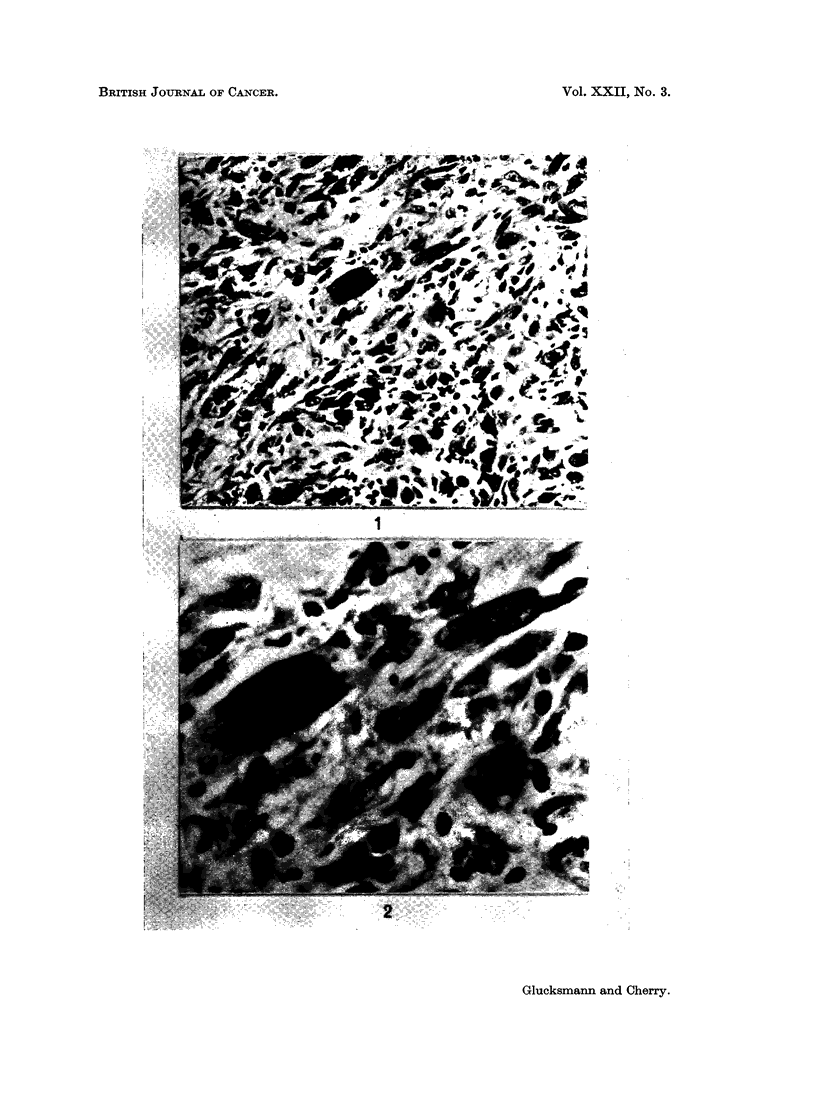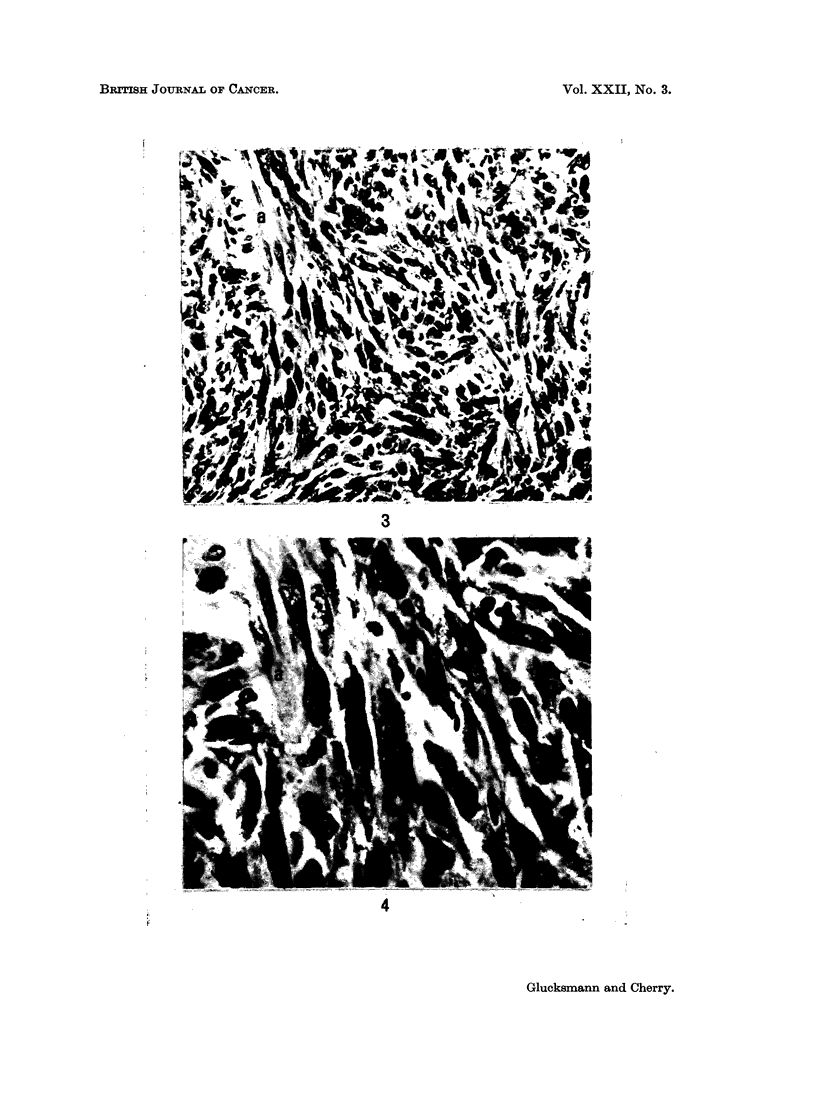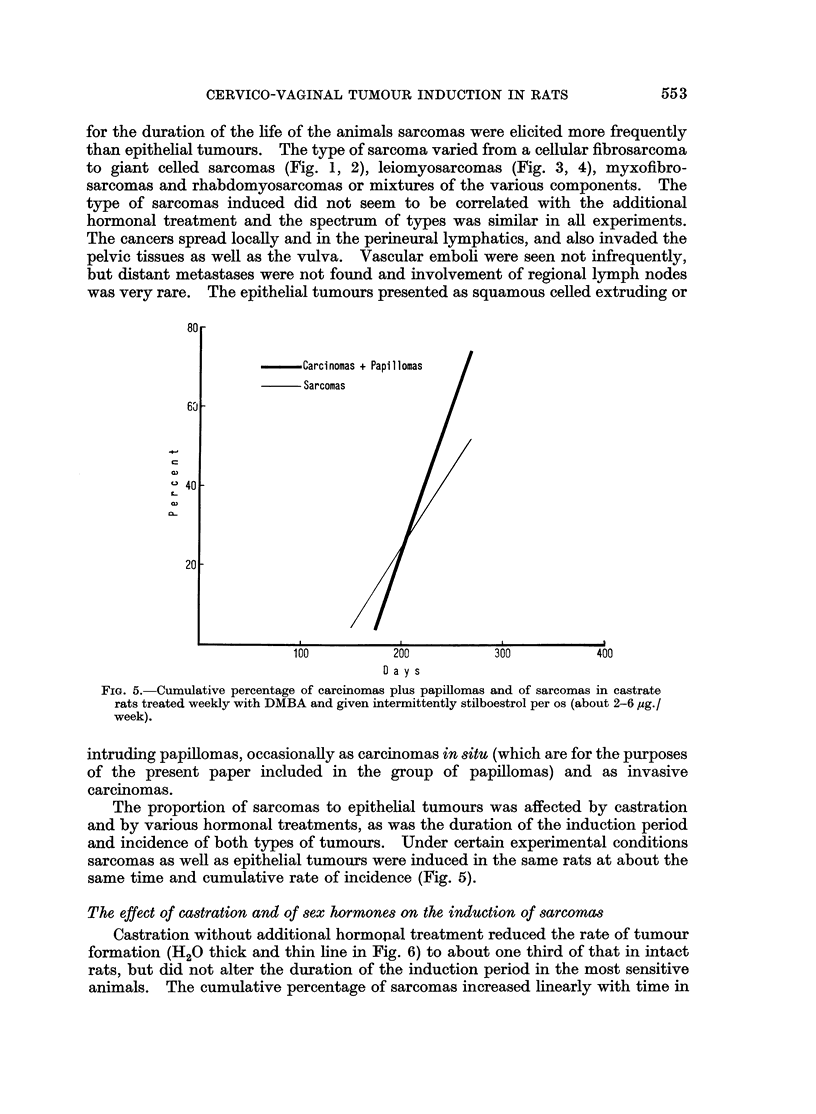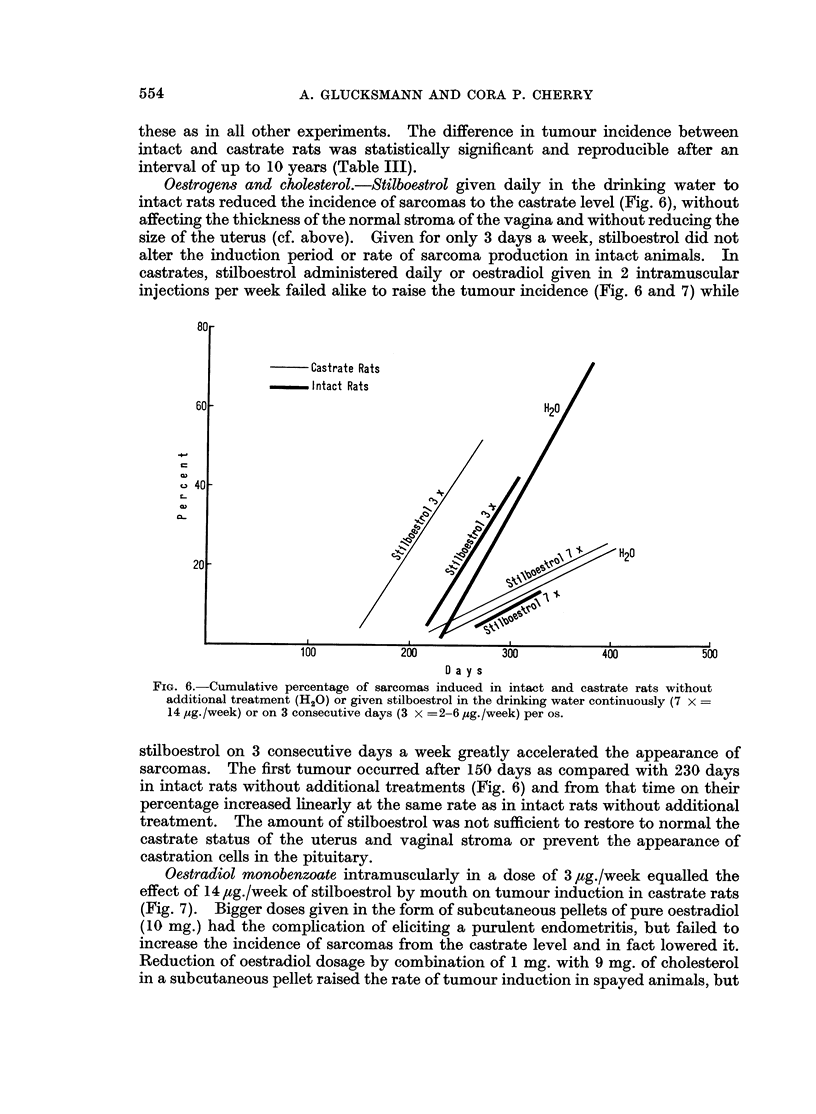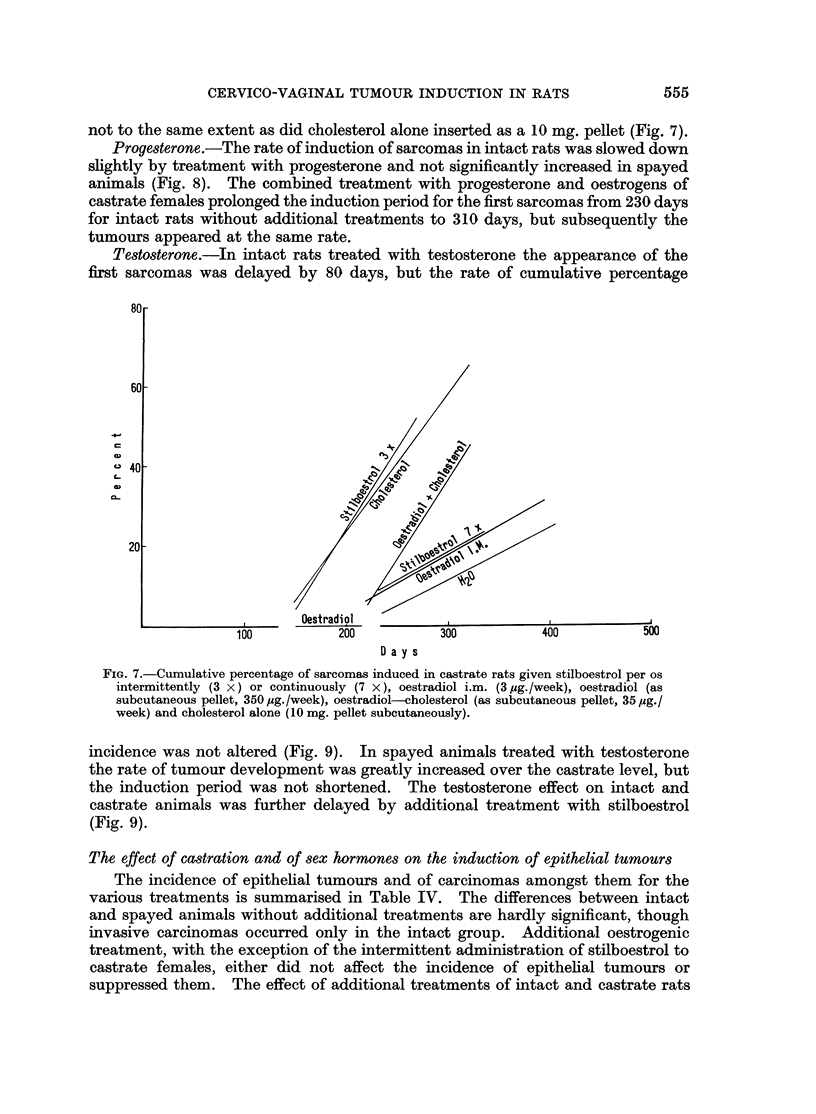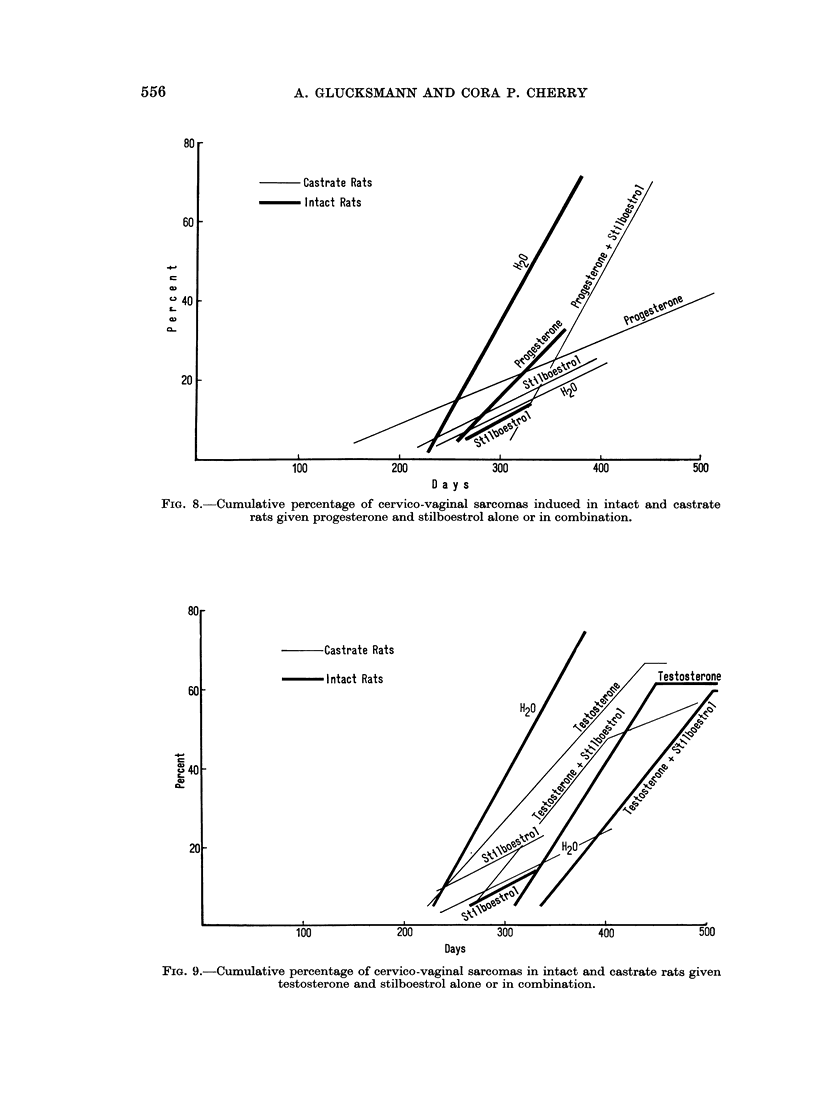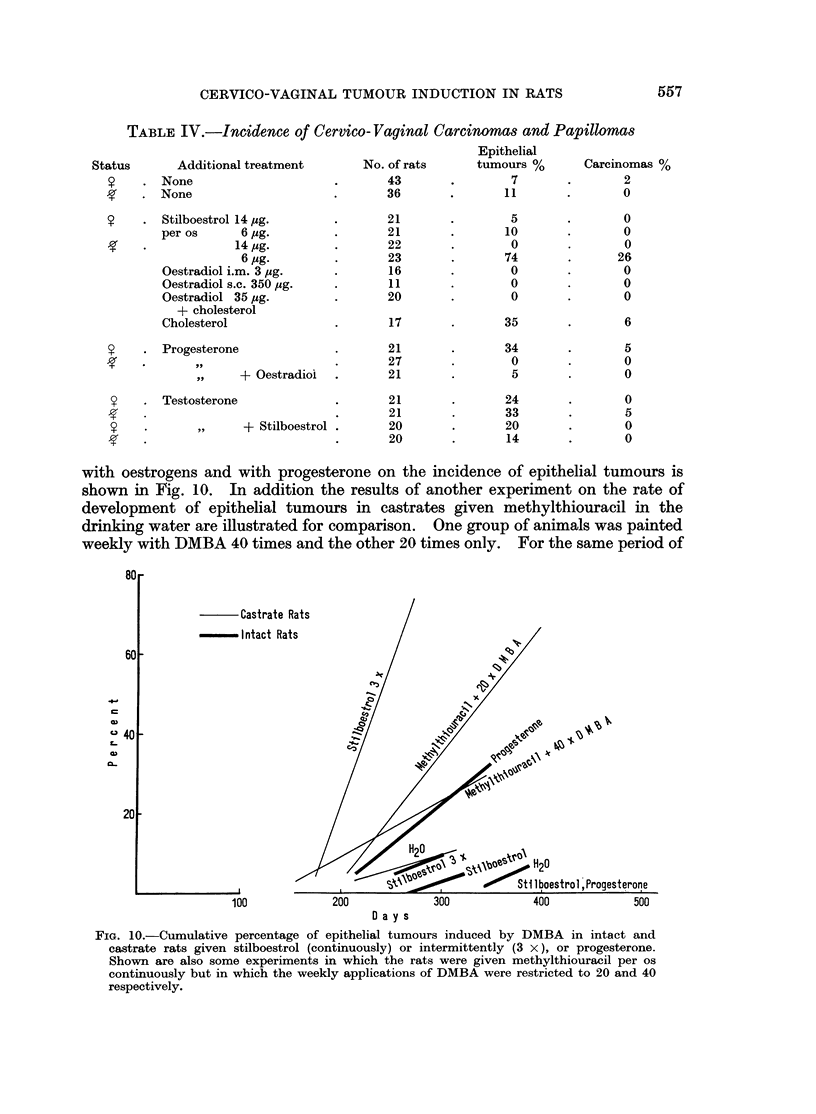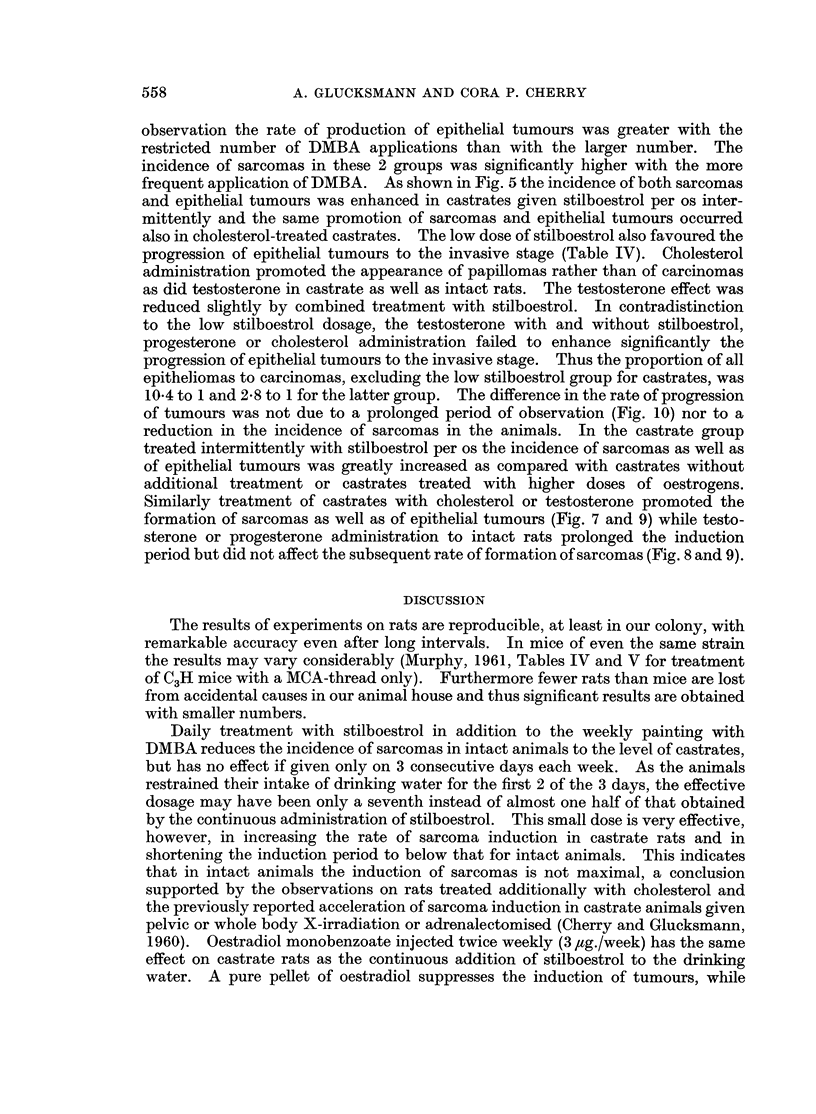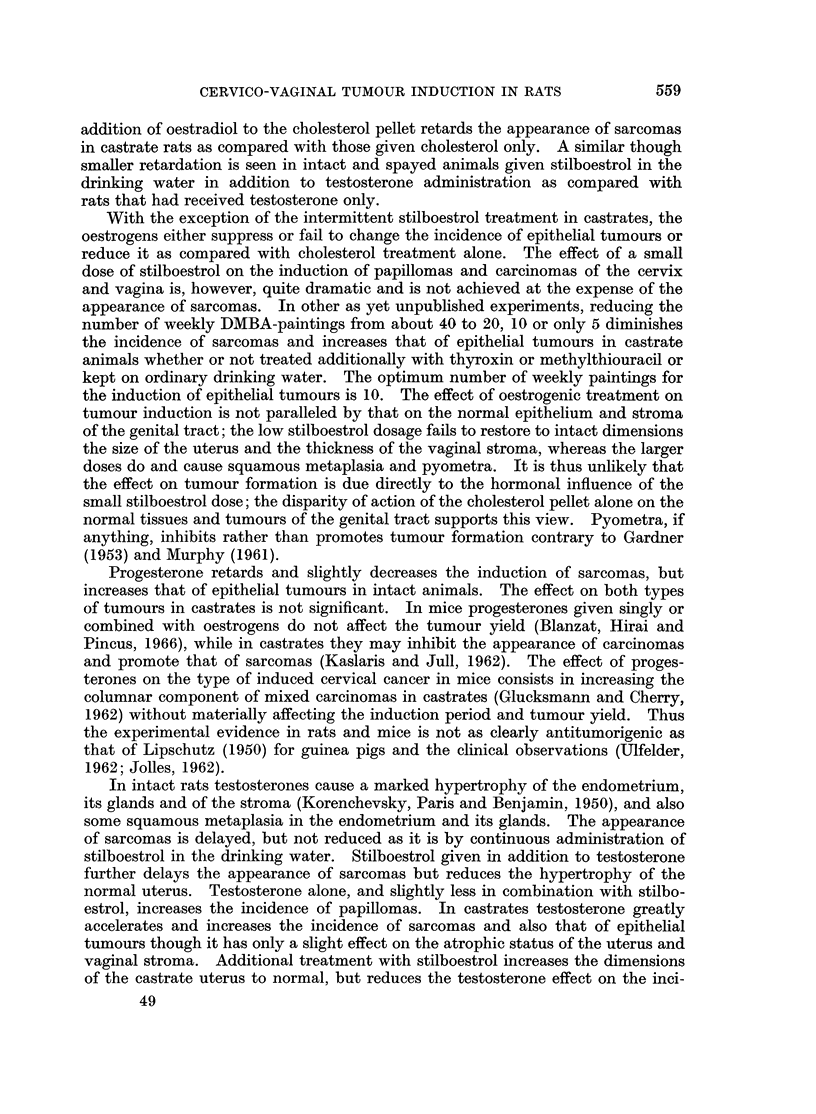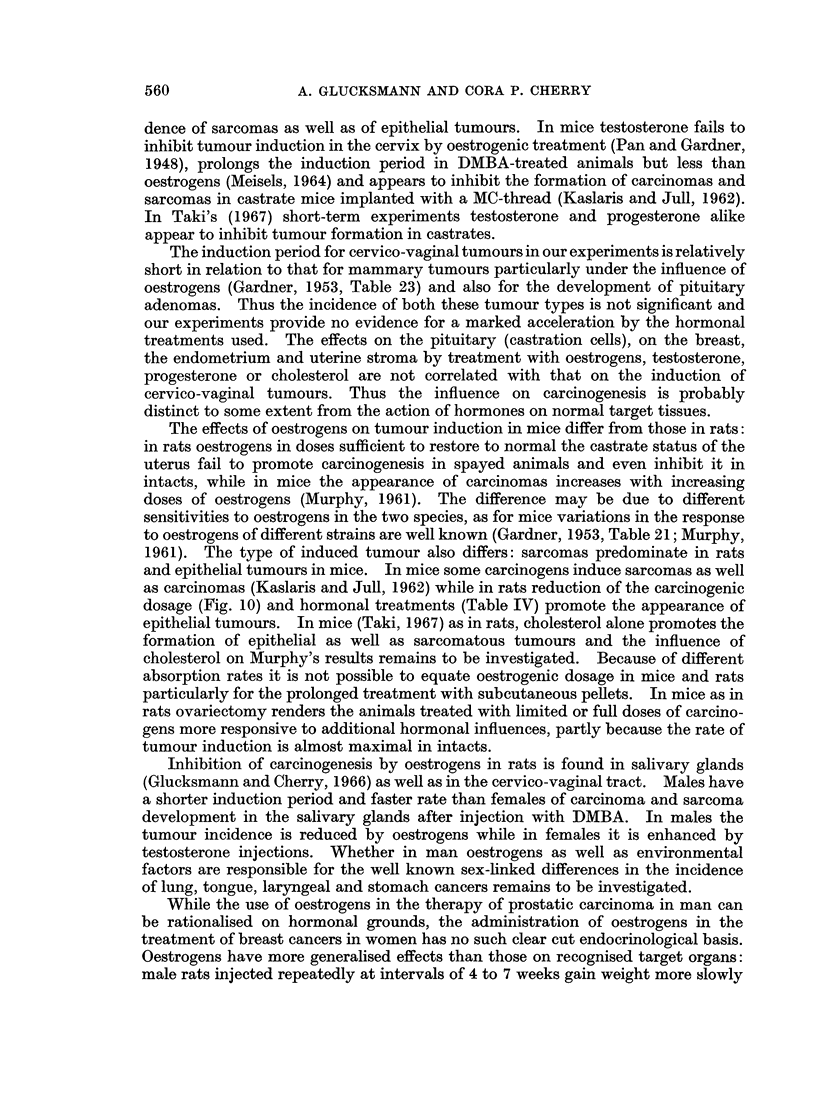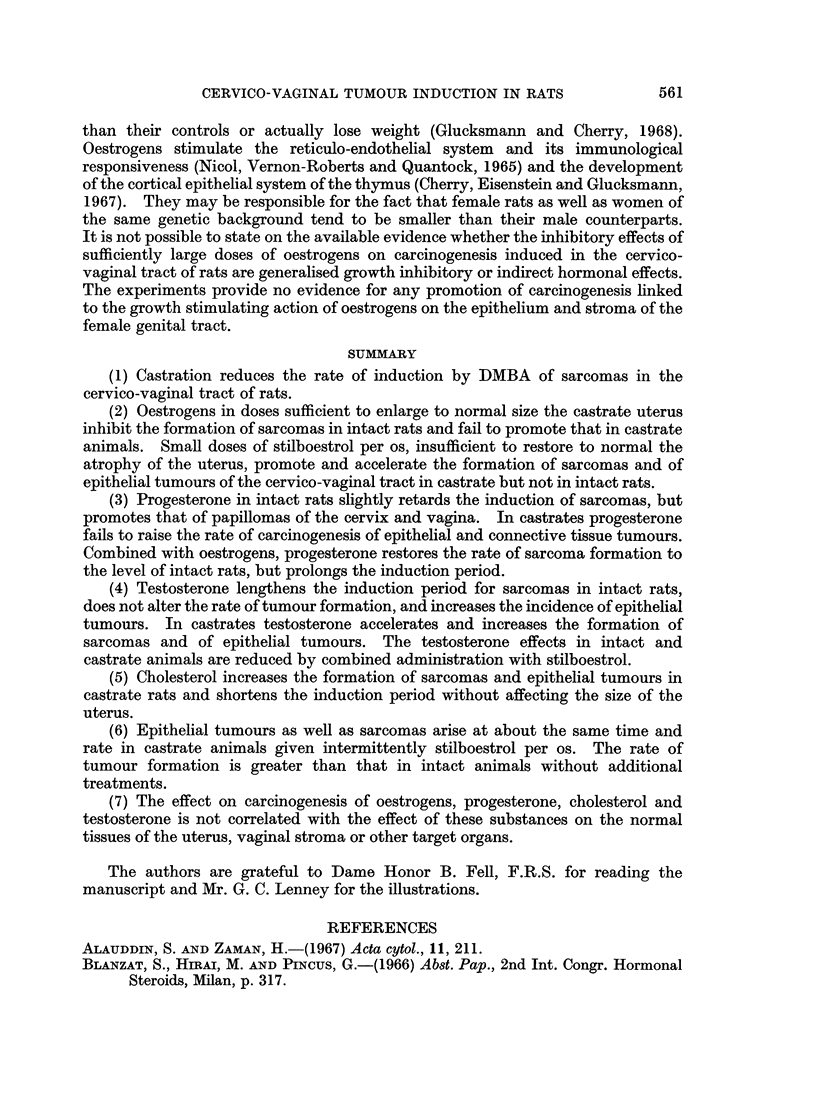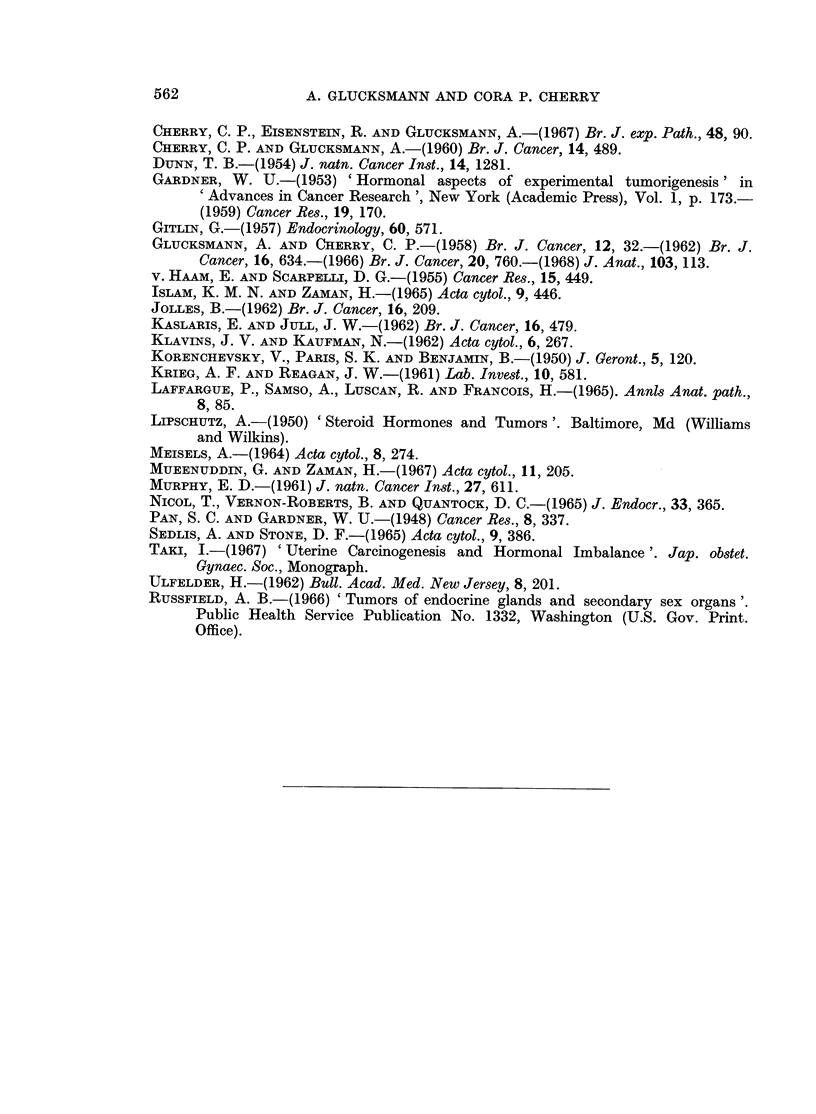# The effect of oestrogens, testosterone and progesterone on the induction of cervico-vaginal tumours in intact and castrate rats.

**DOI:** 10.1038/bjc.1968.65

**Published:** 1968-09

**Authors:** A. Glucksmann, C. P. Cherry

## Abstract

**Images:**


### THE EFFECT OF OESTROGENS, TESTOSTERONE AND PROGESTERONE ON THE INDUCTION OF CERVICO-VAGINAL TUMOURS IN INTACT AND CASTRATE RATS

A. GLUCKSMANN* AND CORA P. CHERRY†

*From the Strangeways Research Laboratory, Cambridge*

Received for publication May 17, 1968

OESTROGENS promote keratinisation in specific organs rather than generally as does vitamin A insufficiency (Gitlin, 1957), suppress the formation of mixed carcinomas of the uterine cervix in mice (Glucksmann and Cherry, 1962), and promote differentiation in the form of keratinisation in cervical tumours of mice (Klavins and Kaufman, 1962). This enhancement of keratinisation by oestrogens might be useful in the treatment of tumours refractory to radiation which often fail to respond to treatment with increased differentiation. This may be particularly useful for the mixed carcinomas of cervix which have given very poor results for both radiotherapy and surgery. On the other hand, oestrogens stimulate proliferation of the epithelium and stroma of the female genital tract, an effect that might be harmful to the patient if produced in tumours at these sites.

The effect of castration, and of additional treatments with oestrogens, progesterone and testosterones have been studied experimentally mainly on mice and have yielded results varying with strain of mice, dosage and type of carcinogen, dosage and type of solvent for the sex hormones. Prolonged administration of oestrogens is followed infrequently by cervical tumours in mice (Gardner, 1959; Murphy, 1961), but in these experiments cholesterol pellets have been used as carriers and cholesterol itself induces cancers. Oestrogen treatment causes septic pyometra which may promote tumour formation, so that oestrogenic hormones may act only indirectly as carcinogenic agents (Gardner, 1953). Administration of oestrogens in combination with chemical carcinogens neither shortens the induction period nor increases the yield of cancers in intact mice (Murphy, 1961; Klavins and Kaufman, 1962; Laffargue, Samso, Luscan and Francois, 1963; Blanzat, Hirai and Pincus, 1966). In castrate mice treated with methylcholanthrene for 4 weeks only, subsequent administration of a diethylstilboestrol-cholesterol pellet has increased the production of tumours and this effect was greater for a 33 % than for a 10 % hormone content of the pellet. The greater incidence of pyometra in mice treated with a 33 % as opposed to a 10 % oestrogenic pellet may account for the higher incidence of carcinomas (Murphy, 1961). Oestrogens do not alter the yield of cancers in spayed mice given full carcinogenic treatment by the thread method or painting. Taki (1967) reports an approximately equal stimulation of cancer production in castrate mice by the additional application of cholesterol alone, oestradiol in cholesterol and oestradiol only, if the treatment and observation period is limited to 5 weeks.

* Gibb Senior Fellow of the British Empire Cancer Campaign for Research.
† Working with a grant from the British Empire Cancer Campaign for Research.

Injections of oestradiol dipropionate have no effect on tumour incidence in castrate mice treated with methylcholanthrene threads (Kaslaris and Jull, 1962; Laffargue *et al.*, 1965). In intact mice Meisels (1964) reports prolongation of the induction period in animals given additional oestradiol injections as compared with those treated with a DMBA-thread only, but no difference in incidence of cervical tumours.

Murphy (1961) finds that castration makes mice more susceptible to limited carcinogenic exposure but detects no difference between castrates and intacts for " full " treatment either by painting of the genital tract or insertion of a thread. Other authors (Krieg and Reagan, 1961; Laffargue *et al.*, 1965; Taki, 1967; Islam and Zaman, 1965; Alauddin and Zaman, 1967; Mueenuddin and Zaman, 1967) report at most an initially faster carcinogenesis in castrate than in intact mice. Mice castrated at 3 to 5 weeks respond with a greater incidence of carcinomas and of sarcomas of the cervico-vaginal tract than those spayed at 4 to 6 months (Kaslaris and Jull, 1962), but no difference is found between virgin mice and those with repeated pregnancies (Sedlis and Stone, 1965). No significant difference in duration of induction time or incidence of carcinomas has been found in intact and castrate mice painted with DMBA in acetone, though the type of resulting carcinoma was altered in spayed as compared with intact animals (Glucksmann and Cherry, 1962). Thus apart from Murphy's finding in castrate mice treated with a carcinogen for 4 weeks only, there is no clear evidence of an enhancing effect of oestrogens on the induction of cervical cancers. The interpretation of Murphy's findings is complicated by his use of cholesterol as vehicle for the oestrogen and by the induction of pyometra with the higher dose of diethylstilboestrol. The rapid response of the cervicovaginal tract in mice to carcinogenic hydrocarbons, makes them unsuitable for experimentation on the effect of castration and additional administration of sex hormones.

In rats marked and highly significant differences in the duration of the induction period and in the incidence of cancers have been reported previously (Glucksmann and Cherry, 1958; Cherry and Glucksmann, 1960), though the majority of the induced tumours are sarcomas. Castration reduces the incidence of sarcomas to one third of that in intact rats and additional treatment with oestrogen or progesterone has failed to increase the tumour incidence significantly. The present communication deals with the effect of castration, that of oestrogenic treatments at different levels of dosage and means of administration, with the effect of testosterone and of progesterone treatment of intact and castrate animals painted once weekly with a potent carcinogen throughout their life span.

### MATERIAL AND METHODS

Hooded rats of the Lister strain, random bred within a closed colony since 1940, were used for the experiments which extended over a period from 1955 to 1966. During this time the incidence of " spontaneous " tumours and other lesions in untreated animals changed as discussed in the relevant section on controls. Some of the experimental procedures were repeated after various intervals and even after 10 years gave essentially the same results.

The rats were housed 7 to a cage, and given water and food pellets *ad libitum*. For certain experiments substances were dissolved and administered in the drinking water. Only rats surviving for at least 120 days after starting an experiment, i.e. the induction period for the first tumours, were considered at risk. A total of 401 animals fell into this category and an additional 76 controls were allowed to complete their natural life span. All animals were examined at weekly intervals and vaginal smears obtained when desirable. Sick individuals and those with clinical or cytological signs of tumours were killed, a post mortem performed and in addition to the organs of the genital tract from ovary to vulva, the following organs were fixed for histological examination: pituitary, thyroid, thymus, salivary glands, lungs, liver, spleen, kidneys, adrenals, intestine, mesenteric and lumbar lymph nodes. The material was fixed in Zenker-acetic, Bouin's or Susa solution, embedded in paraffin after dehydration, sectioned at 6 or 8 $\mu$ depending on the organ and stained with haematoxylin-eosin, van Gieson, carmalum-orange G-aniline blue, Southgate's mucicarmine or the periodic acid-Schiff technique after diastase digestion.

Bilateral ovariectomy was performed with a dorsal approach on rats aged about 4 weeks. The carcinogen 9,10-dimethyl-1,2-benzanthracene (DMBA) as a 1 % solution in acetone was applied at weekly intervals when castrate or intact rats were 2 months old. The solution was administered by means of a cotton wool swab mounted on a thin wire rod which was inserted into the vagina stretched open by dorsal flexion of the tail. By a rotary motion the DMBA was distributed over the cervix, the vaginal walls and inevitably also the vulval region of the introitus. The number of animals thus treated with and without additional hormonal applications is given in Table I. As controls 12 intact and 8 castrate females were painted with acetone only. Like similarly treated mice (Murphy, 1961; v. Haam and Scarpelli, 1955) they failed to produce any tumours in the genital tract.

Additional *oestrogenic* treatment was given by (1) adding 0·1 mg. Stilboestrol B.P. to 1000 ml. of drinking water, thus dosing each rat with about 2 $\mu$g. per day. This addition was given continuously in some experiments (Table I) and in others on 3 days a week (Monday, Tuesday and Wednesday) only. In the intermittent administration rats restricted their water intake for the first 2 days as they did not like the mixture, but were forced by their thirst to drink on the third. In this experiment the weekly dosage was probably only about 2 $\mu$g./week and not the estimated 6 $\mu$g. as given in the Table; (2) 2 intramuscular injections per week of 1·5 $\mu$g. of oestradiol monobenzoate (Organon) in olive oil; (3) subcutaneous implant of a 10 mg. pellet of fused oestradiol monobenzoate (Organon); (4) subcutaneous implant of a pellet consisting of 1 mg. oestradiol monobenzoate and 9 mg. cholesterol (Organon). As controls a group of rats were treated with a 10 mg. pellet of cholesterol.

*Progesterone* (Progestin, Organon) was administered by intramuscular injection in a dose of 1 mg. twice weekly.

*Testosterone propionate* (Ciba) was applied as subcutaneous implant of a 30 mg. pellet, followed by 3 injections of 25 mg. each of microcrystalline Testosterone propionate (Ciba) at intervals of 2 months.

*Combined treatments.*—(a) oestrogens plus progesterone: rats were injected i.m. once weekly with 3 $\mu$g. of oestradiol monobenzoate (Organon) in olive oil and once weekly i.m. with 1 mg. of Progesterone.

(b) oestrogens plus testosterone: Testosterone pellets of 30 mg. were implanted 3 times at intervals of 2 months and stilboestrol (0·1 mg./1000 ml.) was added continuously to the drinking water.

The number of animals at risk and the doses of the additional treatments are given in Table I. The weekly dosage of 6 $\mu$g. for the intermittent stilboestrol administration represents the maximum the animals could have had. In practice the dose may not have exceeded 2 $\mu$g./week.

TABLE I.—*Treatment Groups, Number of Rats at Risk and Weekly Dosages*

| Status | No. of rats | Additional treatment | Form of administration | Dosage per week |
|---|---|---|---|---|
| ♀ | 43 | None | | |
| ♀̸ | 36 | None | | |
| ♀ | 21 | Oestrogens | Stilboestrol in $H_2O$ per os | 14 $\mu$g. |
| | 21 | | *idem* | 6 $\mu$g. |
| ♀̸ | 22 | | *idem* | 14 $\mu$g. |
| | 23 | | *idem* | 6 $\mu$g. |
| | 16 | | Oestradiol monobenzoate, i.m. | 3 $\mu$g. |
| | 11 | | ,, pellet, s.c. | 350 $\mu$g. |
| | 20 | | ,, + cholesterol pellet s.c. | 35 $\mu$g. |
| | 17 | | Cholesterol pellet alone 10 mg. s.c. | |
| ♀ | 21 | Progesterone | Progestin i.m. | 2 mg. |
| ♀̸ | 27 | | *idem* | 2 mg. |
| | 21 | | *idem* | 1 mg. |
| | | | + oestradiol i.m. | 3 mg. |
| ♀ | 21 | Testosterone | Testosterone propionate s.c. | 1·6 mg. |
| ♀̸ | 21 | | *idem* | 1·6 mg. |
| ♀ | 20 | | *idem* | 1·6 mg. |
| | | | + stilboestrol in $H_2O$ | 14 $\mu$g. |
| ♀̸ | 20 | | *idem* | 1·6 mg. |
| | | | + stilboestrol in $H_2O$ | 14 $\mu$g. |

### RESULTS

### *Control animals*

Since hormonal treatments affect tumour incidence in rats and since old rats are subject to endocrine tumours (Russfield, 1966) without additional treatment, it is necessary to analyse tumour incidence and other changes in control animals.

As general controls groups of intact and castrate rats born at different times were allowed to survive their natural life span and the incidence of spontaneous tumours and of other lesions was determined. Special controls were intact or castrate females whose genital tract was painted once weekly with acetone, and another group of intact females whose dorsal skin was painted 4 times at weekly intervals with a 1 % solution of DMBA in acetone. This procedure was adopted to test whether DMBA application influenced the incidence of spontaneous tumours. A further group of 13 females were implanted with a 10 mg. pellet of oestradiol monobenzoate subcutaneously, but received no other treatment. All of them had to be killed after 160 days because of purulent pyometra.

*General controls.*—The first group of 16 intact females born in April 1955 survived for a median period of 744 days (Table II). The relevant post mortem findings were listed as otitis media and labyrinthitis, bronchopneumonia, ileo-caecal lesion which presented as marked dilatation of the ileo-caecal junction with accumulation of faeces, ulceration of the intestinal wall and enlargement and colliquative degeneration of the adjacent lymph nodes. To some extent the ileo-caecal lesion resembles mesenteric disease in mice (Dunn, 1954). The breast tumour was a large fibro-adenoma, the uterine lesion a deciduoma. There were also 2 ovarian tumours in this group, but no leukaemias.

Of 40 females born in October 1964, 20 were left intact and 20 were ovariectomised when 4 weeks old. The first and last dates for death and the median survival period are given in Table II. The castrates had a shorter median survival

TABLE II.—*Morbidity of Control Animals*

| | General controls | | | Special controls | | |
|---|---|---|---|---|---|---|
| Number and sex | 16 ♀ | 20 ♀ | 20 ⚲ | 12 ♀ | 8 ⚲ | 20 ♀ |
| Additional treatment | None | None | None | Acetone painting of vagina | | 4 × DMBA to skin in 3 weeks |
| Born | 1955 | 1964 | 1964 | 1956 | 1956 | 1954 |
| | 29/4 | 20/10 | 20/10 | 25/3 | 27/11 | 12/8 |
| Survival in days: | | | | | | |
| Range | 189–886 | 322–765 | 260–772 | 121–385 | 147–465 | 135–803 |
| Median | 744 | 749 | 675 | 384 | 457 | 470 |
| Number with labyrinthitis | 2 | 2 | 0 | 2 | 2 | 4 |
| Bronchopneumonia | 5 | 9 | 7 | 0 | 0 | 7 |
| Ileo-caecal lesion | 7 | 3 | 3 | 6 | 0 | 14 |
| Leukaemia | 0 | 5 | 5 | 0 | 0 | 0 |
| Breast tumours | 1 | 2 | 0 | 1 | 0 | 2 |
| Uterine tumours | 1 | 1 | 0 | 0 | 0 | 1 |

period than the intact animals. The ileo-caecal lesion occurred with the same frequency in intact and spayed rats and was very much milder than in the group born in 1955: the dilatation was only slight, ulceration less extensive and the adjacent lymph nodes were enlarged but did not undergo colliquative necrosis. In each group 5 leukaemias were found. This lesion started apparently in mesenteric nodes, spread to the lymphatic tissue of the intestine, to the spleen and pancreas, and involved all pelvic and abdominal organs, the lung, mediastinal and cervical nodes and those associated with the thymus which was usually free of the disease. Deposits were found also in the pituitary, ovary and adrenals. In addition one castrate and one intact rat had a haemangioma in a mesenteric node. In the intact animals one of the breast tumours was an adenocarcinoma and the other a fibroadenoma. The uterine tumour was an adenocarcinoma. In addition to the findings listed in Table II the following lesions were noted: pituitary adenomas in 7, solid adenomas of the thyroid in 8, and cortical adenomas of the adrenals in 9 intact rats. Terminal bronchopneumonia was found in most animals, but was pronounced in 9.

In the spayed animals breast and uterine tumours were not seen; pituitary adenomas occurred in 11, solid adenomas of the thyroid in 4, cortical adenomas in 8 and medullary tumours of the adrenal in 2 rats.

*Special controls.*—Acetone painting of the genital tract failed to induce tumours locally in intacts and castrates. The animals were killed when the experimental groups had produced tumours or had died. The breast tumour in the intact group (Table II) was a fibroadenoma. As the animals at death were much younger than the general controls, tumours in the endocrine organs were not yet developed.

In the group treated with DMBA 4 times, the 2 breast tumours were adenocarcinomas, as was the uterine tumour. In addition, a squamous celled carcinoma was found in the mandibular region, i.e. well outside the painted area.

The predominant lesion in the special controls with subcutaneous implants of oestradiol was the purulent endometritis which gave rise to large pelvic and abdominal abscesses. In none of these animals was there any sign of carcinogenesis in the genital tract.

*Comments on the controls.*—In animals born between 1954 and 1956 the incidence of ileo-caecal lesions was fairly high and the disease was extensive, while leukaemias were not found; in those born in 1964 the ileo-caecal lesion occurred less frequently and if present was in a much milder form. On the other hand, the incidence of leukaemias was appreciable and clinically manifest between days 419 and 770. The same findings were made on control males born in 1954 and 1964 respectively: the incidence of ileo-caecal lesions decreased significantly over that period, while that of leukaemias increased.

The incidence of leukaemias in the experiments to be discussed in the present report was much lower than in the controls born at about the same time in 1964. This was due presumably to the fairly long latent period for leukaemias and the much shorter interval between DMBA-painting and the induction of tumours in the genital tract. Thus in the experiments begun in 1963 and lasting well into 1964 only 4 leukaemias were seen in 83 animals and in 1966 only 7 in 86 treated rats. In other experiments, however, carried out at the same time and using L-thyroxin or methylthiouracil as additional treatment, leukaemias occurred as early as 200 days after starting DMBA-painting, i.e. in rats aged 260 days.

Apart from the 2 adenocarcinomas of the uterus and the deciduoma, which are outside the DMBA-treated region, no tumours in the cervico-vaginal tract were found in either the general or special controls.

### *Experimental Animals*

Epithelial tumours, as well as sarcomas, of the cervico-vaginal tract were induced by DMBA-painting and their cumulative percentage increased linearly with time. There were only minor and insignificant deviations from this line. The induction period as well as the rate of tumour production differed for instance between intact and castrate animals without further treatment. Though these differences were statistically highly significant, to test whether the results remained reproducible some of the fundamental experiments were repeated after intervals of 4 to 10 years, occasionally with minor modifications. The results of such repeat experiments are given in Table III. In intact rats painted once weekly with DMBA in 1955 and in 1961, almost identical figures for the induction of sarcomas were obtained. The difference in the incidence of sarcomas in castrates painted once weekly with DMBA in 1956 and another group in 1966 was statistically

TABLE III.—*Cervico-Vaginal Tumours*

| Additional Treatment | None | None | None | None | Stilboestrol per os | Oestradiol i.m. |
|---|---|---|---|---|---|---|
| Date of starting experiment | 1955 24/5 | 1961 14/11 | 1956 28/1 | 1966 20/5 | 1960 17/5 | 1956 28/1 |
| Number at risk | 23 ♀ | 20 ♀ | 15 ⚲ | 21 ⚲ | 22 ⚲ | 16 ⚲ |
| Number with sarcomas | 16 | 15 | 3 | 6 | 5 | 5 |
| Induction period (days) for first | 283 | 184 | 260 | 185 | 232 | 218 |
| and last sarcoma | 381 | 380 | 406 | 315 | 338 | 396 |
| Number with epithelial tumours | 2 | 1 | 0 | 4 | 0 | 0 |

not significant, though the tumours appeared slightly earlier in the repeat than the original experiment. The incidence of papillomas in the second experiment was also slightly higher. The third pair of experiments listed in Table III differed in the form of the oestrogenic treatment: in 1956 the rats were injected twice weekly with oestradiol (3 $\mu$g./week), while in 1960 they received per os about 2 $\mu$g./day of stilboestrol in the drinking water. Again the results were surprisingly similar as regards incidence of sarcomas and epithelial tumours. In fact the cumulative percentage for sarcomas lies on practically the same line (Fig. 7).

For purposes of comparison, in the various graphs the two intact groups were averaged and plotted and the same was done for the castrates.

*Effects of castration and of hormonal treatments*

Castration caused atrophy of the stroma of the vagina and uterus and quantitative data about this reduction were given in a previous paper (Glucksmann and Cherry, 1958). The vaginal epithelium was reduced to one to two layers of mucus-secreting cuboidal or columnar cells. Normal tissues in animals in which acetone or DMBA in acetone was applied to the vagina reacted alike to hormones and differed in their morphology from similarly treated animals that were not given hormones.

*Stilboestrol* induced a high cornifying vaginal and cervical epithelium in intact animals, did not greatly affect the vaginal stroma, caused only slight hyperplasia of the uterus and in less than 10 % of the rats treated with the higher dosage (14 $\mu$g./week) produced a purulent endometritis. Squamous metaplasia of the endometrial epithelium and glands was not observed, but a deciduoma was found in one of the rats. In castrates the higher dosage of stilboestrol restored the thickness of the vaginal stroma to that of intact animals, induced cornification in the stratified vaginal and cervical epithelium, increased the uterine diameter to intact size in 18 of 22 rats treated with the higher dosage and to about 90 % normal size in the remaining animals. With the lower dosage the uterus was only half the normal size in 56 % of rats and three quarters in 44 %. The vaginal and cervical epithelium were of cornifying stratified type and the width of the vaginal stroma was greater than in castrates.

*Oestradiol* at all 3 dosages in castrate rats restored the thickness of the vaginal stroma to intact level, induced a high keratinising squamous epithelium in cervix and vagina and enlargement of the uterus. The incidence of squamous metaplasia in endometrial glands and epithelium increased with dosage from 6 % at 3 $\mu$g./week to 65 % at 35 $\mu$g./week. In castrate rats given 350 $\mu$g./week 54 % had squamous metaplasia, while the epithelium in the rest was destroyed by an intense purulent reaction. In the control rats of this group treated with a *cholesterol* pellet alone the castrate status of the vaginal stroma and epithelium and of the uterus was not altered.

*Progesterone* given to intact animals did not change the thickness of the vaginal stroma, induced mucification in a high columnar epithelium of the cervix while the DMBA-treated vaginal epithelium was stratified. In about a third of the animals the uterus was only 90 % of the normal size. The endometrial epithelium was high and columnar, but the glands showed little development. In only 19 % of castrate animals was the uterus restored to normal size, while the others remained in the castrate state. Similarly the vaginal stroma remained of castrate type and thickness.

*Testosterone* administration to intact animals caused enlargement of the uterus with high columnar epithelium and marked hyperplasia of glands. In almost 50 % of the treated rats evidence of squamous metaplasia in the endometrial glands or the endometrial lining was found. The cervical epithelium tended to be of columnar mucifying type, while DMBA-treated vaginal epithelium was stratified and cornified. In castrate animals treated with testosterone only about 10 % had a uterine diameter of normal size, while in the rest it was of castrate dimensions. The vaginal stroma remained reduced in thickness. Additional treatment with stilboestrol in the drinking water raised the proportion of spayed rats with normal sized uteri to 40 %, and also increased the thickness of the vaginal stroma. In intact animals the combined treatment with stilboestrol and testosterone caused hyperplasia of the uterus but to a lesser extent than did testosterone alone. Only 30 % of the animals showed squamous metaplasia of the endometrial glands and in 20 % there was some decidual reaction in the endometrial stroma. The cervical epithelium tended to be of the columnar mucifying type.

In intact as well as castrate rats treated with testosterone as also in those given additionally stilboestrol, the median tissue of the clitoris was transformed into a calcified and in places ossified rod of fibrocartilage.

Castration caused hypertrophy of gonadotrophs and the appearance of castration cells in the pituitary followed later by the appearance of adenomas. Stilboestrol by mouth, even when given continuously, failed to prevent the formation of castration cells. In castrates treated with a subcutaneous oestradiol-cholesterol pellet and in those given progesterone and oestradiol by injection, the gonadotrophs were hypertrophic and hyperplastic but castration cells were not found during the experiment. Progesterone alone did not prevent the occurrence of castration cells. After testosterone treatment the castration cells were not as prominent as after stilboestrol administration. The combined treatment with testosterone and stilboestrol somewhat delayed the appearance of castration cells. Though as compared with the castrate controls, the incidence of pituitary adenomas was reduced by oestrogenic and testosterone treatment, this effect was probably due to the shorter survival period of the DMBA-treated animals as compared with the controls as pituitary adenomas had a long induction period in castrate rats.

Testosterone treatment reduced the incidence of growing follicles and corpora lutea and increased that of atretic structures. The other treatments did not greatly affect the appearance of follicles and corpora lutea.

*The induction of cervico-vaginal tumours*

Neither acetone painting alone nor the implantation of oestradiol pellets induced any tumours in the cervico-vaginal tract. DMBA-painting was necessary to elicit tumour formation which resulted in the formation of sarcomas in the subepithelial vaginal tissue or of papillomas, intra-epithelial and invasive carcinomas of the vaginal or cervical epithelium. With weekly applications of DMBA

---

EXPLANATION OF PLATES

FIG. 1 and 2.—A cellular sarcoma of the vagina with giant cells induced in an intact rat treated weekly with DMBA and 2 injections of Progestin per week. × 265 and × 680.

FIG. 3 and 4.—A leiomyosarcomatous component in a cellular sarcoma of the vagina in an intact rat treated weekly with DMBA and 2 injections of Progestin per week. (a) normal smooth muscle fibres. × 265 and × 680.

1

2

3

4

Glucksmann and Cherry.

for the duration of the life of the animals sarcomas were elicited more frequently than epithelial tumours. The type of sarcoma varied from a cellular fibrosarcoma to giant celled sarcomas (Fig. 1, 2), leiomyosarcomas (Fig. 3, 4), myxofibrosarcomas and rhabdomyosarcomas or mixtures of the various components. The type of sarcomas induced did not seem to be correlated with the additional hormonal treatment and the spectrum of types was similar in all experiments. The cancers spread locally and in the perineural lymphatics, and also invaded the pelvic tissues as well as the vulva. Vascular emboli were seen not infrequently, but distant metastases were not found and involvement of regional lymph nodes was very rare. The epithelial tumours presented as squamous celled extruding or

FIG. 5.—Cumulative percentage of carcinomas plus papillomas and of sarcomas in castrate rats treated weekly with DMBA and given intermittently stilboestrol per os (about 2–6 μg./week).

intruding papillomas, occasionally as carcinomas *in situ* (which are for the purposes of the present paper included in the group of papillomas) and as invasive carcinomas.

The proportion of sarcomas to epithelial tumours was affected by castration and by various hormonal treatments, as was the duration of the induction period and incidence of both types of tumours. Under certain experimental conditions sarcomas as well as epithelial tumours were induced in the same rats at about the same time and cumulative rate of incidence (Fig. 5).

*The effect of castration and of sex hormones on the induction of sarcomas*

Castration without additional hormonal treatment reduced the rate of tumour formation ($H_2O$ thick and thin line in Fig. 6) to about one third of that in intact rats, but did not alter the duration of the induction period in the most sensitive animals. The cumulative percentage of sarcomas increased linearly with time in these as in all other experiments. The difference in tumour incidence between intact and castrate rats was statistically significant and reproducible after an interval of up to 10 years (Table III).

*Oestrogens and cholesterol.—Stilboestrol* given daily in the drinking water to intact rats reduced the incidence of sarcomas to the castrate level (Fig. 6), without affecting the thickness of the normal stroma of the vagina and without reducing the size of the uterus (cf. above). Given for only 3 days a week, stilboestrol did not alter the induction period or rate of sarcoma production in intact animals. In castrates, stilboestrol administered daily or oestradiol given in 2 intramuscular injections per week failed alike to raise the tumour incidence (Fig. 6 and 7) while

Fig. 6.—Cumulative percentage of sarcomas induced in intact and castrate rats without additional treatment ($H_2O$) or given stilboestrol in the drinking water continuously (7 × = 14 μg./week) or on 3 consecutive days (3 × = 2–6 μg./week) per os.

stilboestrol on 3 consecutive days a week greatly accelerated the appearance of sarcomas. The first tumour occurred after 150 days as compared with 230 days in intact rats without additional treatments (Fig. 6) and from that time on their percentage increased linearly at the same rate as in intact rats without additional treatment. The amount of stilboestrol was not sufficient to restore to normal the castrate status of the uterus and vaginal stroma or prevent the appearance of castration cells in the pituitary.

*Oestradiol monobenzoate* intramuscularly in a dose of 3 μg./week equalled the effect of 14 μg./week of stilboestrol by mouth on tumour induction in castrate rats (Fig. 7). Bigger doses given in the form of subcutaneous pellets of pure oestradiol (10 mg.) had the complication of eliciting a purulent endometritis, but failed to increase the incidence of sarcomas from the castrate level and in fact lowered it. Reduction of oestradiol dosage by combination of 1 mg. with 9 mg. of cholesterol in a subcutaneous pellet raised the rate of tumour induction in spayed animals, but not to the same extent as did cholesterol alone inserted as a 10 mg. pellet (Fig. 7).

*Progesterone.*—The rate of induction of sarcomas in intact rats was slowed down slightly by treatment with progesterone and not significantly increased in spayed animals (Fig. 8). The combined treatment with progesterone and oestrogens of castrate females prolonged the induction period for the first sarcomas from 230 days for intact rats without additional treatments to 310 days, but subsequently the tumours appeared at the same rate.

*Testosterone.*—In intact rats treated with testosterone the appearance of the first sarcomas was delayed by 80 days, but the rate of cumulative percentage

FIG. 7.—Cumulative percentage of sarcomas induced in castrate rats given stilboestrol per os intermittently (3 ×) or continuously (7 ×), oestradiol i.m. (3 μg./week), oestradiol (as subcutaneous pellet, 350 μg./week), oestradiol—cholesterol (as subcutaneous pellet, 35 μg./week) and cholesterol alone (10 mg. pellet subcutaneously).

incidence was not altered (Fig. 9). In spayed animals treated with testosterone the rate of tumour development was greatly increased over the castrate level, but the induction period was not shortened. The testosterone effect on intact and castrate animals was further delayed by additional treatment with stilboestrol (Fig. 9).

*The effect of castration and of sex hormones on the induction of epithelial tumours*

The incidence of epithelial tumours and of carcinomas amongst them for the various treatments is summarised in Table IV. The differences between intact and spayed animals without additional treatments are hardly significant, though invasive carcinomas occurred only in the intact group. Additional oestrogenic treatment, with the exception of the intermittent administration of stilboestrol to castrate females, either did not affect the incidence of epithelial tumours or suppressed them. The effect of additional treatments of intact and castrate rats

FIG. 8.—Cumulative percentage of cervico-vaginal sarcomas induced in intact and castrate rats given progesterone and stilboestrol alone or in combination.

FIG. 9.—Cumulative percentage of cervico-vaginal sarcomas in intact and castrate rats given testosterone and stilboestrol alone or in combination.

TABLE IV.—*Incidence of Cervico-Vaginal Carcinomas and Papillomas*

| Status | Additional treatment | No. of rats | Epithelial tumours % | Carcinomas % |
|---|---|---|---|---|
| ♀ | None | 43 | 7 | 2 |
| ♀̸ | None | 36 | 11 | 0 |
| ♀ | Stilboestrol 14 µg. | 21 | 5 | 0 |
| | per os 6 µg. | 21 | 10 | 0 |
| ♀̸ | 14 µg. | 22 | 0 | 0 |
| | 6 µg. | 23 | 74 | 26 |
| | Oestradiol i.m. 3 µg. | 16 | 0 | 0 |
| | Oestradiol s.c. 350 µg. | 11 | 0 | 0 |
| | Oestradiol 35 µg. + cholesterol | 20 | 0 | 0 |
| | Cholesterol | 17 | 35 | 6 |
| ♀ | Progesterone | 21 | 34 | 5 |
| ♀̸ | ,, | 27 | 0 | 0 |
| | ,, + Oestradiol | 21 | 5 | 0 |
| ♀ | Testosterone | 21 | 24 | 0 |
| ♀̸ | | 21 | 33 | 5 |
| ♀ | ,, + Stilboestrol | 20 | 20 | 0 |
| ♀̸ | | 20 | 14 | 0 |

with oestrogens and with progesterone on the incidence of epithelial tumours is shown in Fig. 10. In addition the results of another experiment on the rate of development of epithelial tumours in castrates given methylthiouracil in the drinking water are illustrated for comparison. One group of animals was painted weekly with DMBA 40 times and the other 20 times only. For the same period of

FIG. 10.—Cumulative percentage of epithelial tumours induced by DMBA in intact and castrate rats given stilboestrol (continuously) or intermittently (3 ×), or progesterone. Shown are also some experiments in which the rats were given methylthiouracil per os continuously but in which the weekly applications of DMBA were restricted to 20 and 40 respectively.

observation the rate of production of epithelial tumours was greater with the restricted number of DMBA applications than with the larger number. The incidence of sarcomas in these 2 groups was significantly higher with the more frequent application of DMBA. As shown in Fig. 5 the incidence of both sarcomas and epithelial tumours was enhanced in castrates given stilboestrol per os intermittently and the same promotion of sarcomas and epithelial tumours occurred also in cholesterol-treated castrates. The low dose of stilboestrol also favoured the progression of epithelial tumours to the invasive stage (Table IV). Cholesterol administration promoted the appearance of papillomas rather than of carcinomas as did testosterone in castrate as well as intact rats. The testosterone effect was reduced slightly by combined treatment with stilboestrol. In contradistinction to the low stilboestrol dosage, the testosterone with and without stilboestrol, progesterone or cholesterol administration failed to enhance significantly the progression of epithelial tumours to the invasive stage. Thus the proportion of all epitheliomas to carcinomas, excluding the low stilboestrol group for castrates, was 10·4 to 1 and 2·8 to 1 for the latter group. The difference in the rate of progression of tumours was not due to a prolonged period of observation (Fig. 10) nor to a reduction in the incidence of sarcomas in the animals. In the castrate group treated intermittently with stilboestrol per os the incidence of sarcomas as well as of epithelial tumours was greatly increased as compared with castrates without additional treatment or castrates treated with higher doses of oestrogens. Similarly treatment of castrates with cholesterol or testosterone promoted the formation of sarcomas as well as of epithelial tumours (Fig. 7 and 9) while testosterone or progesterone administration to intact rats prolonged the induction period but did not affect the subsequent rate of formation of sarcomas (Fig. 8 and 9).

### DISCUSSION

The results of experiments on rats are reproducible, at least in our colony, with remarkable accuracy even after long intervals. In mice of even the same strain the results may vary considerably (Murphy, 1961, Tables IV and V for treatment of $C_3H$ mice with a MCA-thread only). Furthermore fewer rats than mice are lost from accidental causes in our animal house and thus significant results are obtained with smaller numbers.

Daily treatment with stilboestrol in addition to the weekly painting with DMBA reduces the incidence of sarcomas in intact animals to the level of castrates, but has no effect if given only on 3 consecutive days each week. As the animals restrained their intake of drinking water for the first 2 of the 3 days, the effective dosage may have been only a seventh instead of almost one half of that obtained by the continuous administration of stilboestrol. This small dose is very effective, however, in increasing the rate of sarcoma induction in castrate rats and in shortening the induction period to below that for intact animals. This indicates that in intact animals the induction of sarcomas is not maximal, a conclusion supported by the observations on rats treated additionally with cholesterol and the previously reported acceleration of sarcoma induction in castrate animals given pelvic or whole body X-irradiation or adrenalectomised (Cherry and Glucksmann, 1960). Oestradiol monobenzoate injected twice weekly (3 $\mu$g./week) has the same effect on castrate rats as the continuous addition of stilboestrol to the drinking water. A pure pellet of oestradiol suppresses the induction of tumours, while addition of oestradiol to the cholesterol pellet retards the appearance of sarcomas in castrate rats as compared with those given cholesterol only. A similar though smaller retardation is seen in intact and spayed animals given stilboestrol in the drinking water in addition to testosterone administration as compared with rats that had received testosterone only.

With the exception of the intermittent stilboestrol treatment in castrates, the oestrogens either suppress or fail to change the incidence of epithelial tumours or reduce it as compared with cholesterol treatment alone. The effect of a small dose of stilboestrol on the induction of papillomas and carcinomas of the cervix and vagina is, however, quite dramatic and is not achieved at the expense of the appearance of sarcomas. In other as yet unpublished experiments, reducing the number of weekly DMBA-paintings from about 40 to 20, 10 or only 5 diminishes the incidence of sarcomas and increases that of epithelial tumours in castrate animals whether or not treated additionally with thyroxin or methylthiouracil or kept on ordinary drinking water. The optimum number of weekly paintings for the induction of epithelial tumours is 10. The effect of oestrogenic treatment on tumour induction is not paralleled by that on the normal epithelium and stroma of the genital tract; the low stilboestrol dosage fails to restore to intact dimensions the size of the uterus and the thickness of the vaginal stroma, whereas the larger doses do and cause squamous metaplasia and pyometra. It is thus unlikely that the effect on tumour formation is due directly to the hormonal influence of the small stilboestrol dose; the disparity of action of the cholesterol pellet alone on the normal tissues and tumours of the genital tract supports this view. Pyometra, if anything, inhibits rather than promotes tumour formation contrary to Gardner (1953) and Murphy (1961).

Progesterone retards and slightly decreases the induction of sarcomas, but increases that of epithelial tumours in intact animals. The effect on both types of tumours in castrates is not significant. In mice progesterones given singly or combined with oestrogens do not affect the tumour yield (Blanzat, Hirai and Pincus, 1966), while in castrates they may inhibit the appearance of carcinomas and promote that of sarcomas (Kaslaris and Jull, 1962). The effect of progesterones on the type of induced cervical cancer in mice consists in increasing the columnar component of mixed carcinomas in castrates (Glucksmann and Cherry, 1962) without materially affecting the induction period and tumour yield. Thus the experimental evidence in rats and mice is not as clearly antitumorigenic as that of Lipschutz (1950) for guinea pigs and the clinical observations (Ulfelder, 1962; Jolles, 1962).

In intact rats testosterones cause a marked hypertrophy of the endometrium, its glands and of the stroma (Korenchevsky, Paris and Benjamin, 1950), and also some squamous metaplasia in the endometrium and its glands. The appearance of sarcomas is delayed, but not reduced as it is by continuous administration of stilboestrol in the drinking water. Stilboestrol given in addition to testosterone further delays the appearance of sarcomas but reduces the hypertrophy of the normal uterus. Testosterone alone, and slightly less in combination with stilboestrol, increases the incidence of papillomas. In castrates testosterone greatly accelerates and increases the incidence of sarcomas and also that of epithelial tumours though it has only a slight effect on the atrophic status of the uterus and vaginal stroma. Additional treatment with stilboestrol increases the dimensions of the castrate uterus to normal, but reduces the testosterone effect on the incidence of sarcomas as well as of epithelial tumours. In mice testosterone fails to inhibit tumour induction in the cervix by oestrogenic treatment (Pan and Gardner, 1948), prolongs the induction period in DMBA-treated animals but less than oestrogens (Meisels, 1964) and appears to inhibit the formation of carcinomas and sarcomas in castrate mice implanted with a MC-thread (Kaslaris and Jull, 1962). In Taki's (1967) short-term experiments testosterone and progesterone alike appear to inhibit tumour formation in castrates.

The induction period for cervico-vaginal tumours in our experiments is relatively short in relation to that for mammary tumours particularly under the influence of oestrogens (Gardner, 1953, Table 23) and also for the development of pituitary adenomas. Thus the incidence of both these tumour types is not significant and our experiments provide no evidence for a marked acceleration by the hormonal treatments used. The effects on the pituitary (castration cells), on the breast, the endometrium and uterine stroma by treatment with oestrogens, testosterone, progesterone or cholesterol are not correlated with that on the induction of cervico-vaginal tumours. Thus the influence on carcinogenesis is probably distinct to some extent from the action of hormones on normal target tissues.

The effects of oestrogens on tumour induction in mice differ from those in rats: in rats oestrogens in doses sufficient to restore to normal the castrate status of the uterus fail to promote carcinogenesis in spayed animals and even inhibit it in intacts, while in mice the appearance of carcinomas increases with increasing doses of oestrogens (Murphy, 1961). The difference may be due to different sensitivities to oestrogens in the two species, as for mice variations in the response to oestrogens of different strains are well known (Gardner, 1953, Table 21; Murphy, 1961). The type of induced tumour also differs: sarcomas predominate in rats and epithelial tumours in mice. In mice some carcinogens induce sarcomas as well as carcinomas (Kaslaris and Jull, 1962) while in rats reduction of the carcinogenic dosage (Fig. 10) and hormonal treatments (Table IV) promote the appearance of epithelial tumours. In mice (Taki, 1967) as in rats, cholesterol alone promotes the formation of epithelial as well as sarcomatous tumours and the influence of cholesterol on Murphy's results remains to be investigated. Because of different absorption rates it is not possible to equate oestrogenic dosage in mice and rats particularly for the prolonged treatment with subcutaneous pellets. In mice as in rats ovariectomy renders the animals treated with limited or full doses of carcinogens more responsive to additional hormonal influences, partly because the rate of tumour induction is almost maximal in intacts.

Inhibition of carcinogenesis by oestrogens in rats is found in salivary glands (Glucksmann and Cherry, 1966) as well as in the cervico-vaginal tract. Males have a shorter induction period and faster rate than females of carcinoma and sarcoma development in the salivary glands after injection with DMBA. In males the tumour incidence is reduced by oestrogens while in females it is enhanced by testosterone injections. Whether in man oestrogens as well as environmental factors are responsible for the well known sex-linked differences in the incidence of lung, tongue, laryngeal and stomach cancers remains to be investigated.

While the use of oestrogens in the therapy of prostatic carcinoma in man can be rationalised on hormonal grounds, the administration of oestrogens in the treatment of breast cancers in women has no such clear cut endocrinological basis. Oestrogens have more generalised effects than those on recognised target organs: male rats injected repeatedly at intervals of 4 to 7 weeks gain weight more slowly than their controls or actually lose weight (Glucksmann and Cherry, 1968). Oestrogens stimulate the reticulo-endothelial system and its immunological responsiveness (Nicol, Vernon-Roberts and Quantock, 1965) and the development of the cortical epithelial system of the thymus (Cherry, Eisenstein and Glucksmann, 1967). They may be responsible for the fact that female rats as well as women of the same genetic background tend to be smaller than their male counterparts. It is not possible to state on the available evidence whether the inhibitory effects of sufficiently large doses of oestrogens on carcinogenesis induced in the cervico-vaginal tract of rats are generalised growth inhibitory or indirect hormonal effects. The experiments provide no evidence for any promotion of carcinogenesis linked to the growth stimulating action of oestrogens on the epithelium and stroma of the female genital tract.

### SUMMARY

(1) Castration reduces the rate of induction by DMBA of sarcomas in the cervico-vaginal tract of rats.

(2) Oestrogens in doses sufficient to enlarge to normal size the castrate uterus inhibit the formation of sarcomas in intact rats and fail to promote that in castrate animals. Small doses of stilboestrol per os, insufficient to restore to normal the atrophy of the uterus, promote and accelerate the formation of sarcomas and of epithelial tumours of the cervico-vaginal tract in castrate but not in intact rats.

(3) Progesterone in intact rats slightly retards the induction of sarcomas, but promotes that of papillomas of the cervix and vagina. In castrates progesterone fails to raise the rate of carcinogenesis of epithelial and connective tissue tumours. Combined with oestrogens, progesterone restores the rate of sarcoma formation to the level of intact rats, but prolongs the induction period.

(4) Testosterone lengthens the induction period for sarcomas in intact rats, does not alter the rate of tumour formation, and increases the incidence of epithelial tumours. In castrates testosterone accelerates and increases the formation of sarcomas and of epithelial tumours. The testosterone effects in intact and castrate animals are reduced by combined administration with stilboestrol.

(5) Cholesterol increases the formation of sarcomas and epithelial tumours in castrate rats and shortens the induction period without affecting the size of the uterus.

(6) Epithelial tumours as well as sarcomas arise at about the same time and rate in castrate animals given intermittently stilboestrol per os. The rate of tumour formation is greater than that in intact animals without additional treatments.

(7) The effect on carcinogenesis of oestrogens, progesterone, cholesterol and testosterone is not correlated with the effect of these substances on the normal tissues of the uterus, vaginal stroma or other target organs.

The authors are grateful to Dame Honor B. Fell, F.R.S. for reading the manuscript and Mr. G. C. Lenney for the illustrations.

### REFERENCES

ALAUDDIN, S. AND ZAMAN, H.—(1967) *Acta cytol.*, **11**, 211.

BLANZAT, S., HIRAI, M. AND PINCUS, G.—(1966) *Abst. Pap.*, 2nd Int. Congr. Hormonal Steroids, Milan, p. 317.

Cherry, C. P., Eisenstein, R. and Glucksmann, A.—(1967) *Br. J. exp. Path.*, **48**, 90.
Cherry, C. P. and Glucksmann, A.—(1960) *Br. J. Cancer*, **14**, 489.
Dunn, T. B.—(1954) *J. natn. Cancer Inst.*, **14**, 1281.
Gardner, W. U.—(1953) ' Hormonal aspects of experimental tumorigenesis ' in ' Advances in Cancer Research ', New York (Academic Press), Vol. 1, p. 173.—(1959) *Cancer Res.*, **19**, 170.
Gitlin, G.—(1957) *Endocrinology*, **60**, 571.
Glucksmann, A. and Cherry, C. P.—(1958) *Br. J. Cancer*, **12**, 32.—(1962) *Br. J. Cancer*, **16**, 634.—(1966) *Br. J. Cancer*, **20**, 760.—(1968) *J. Anat.*, **103**, 113.
v. Haam, E. and Scarpelli, D. G.—(1955) *Cancer Res.*, **15**, 449.
Islam, K. M. N. and Zaman, H.—(1965) *Acta cytol.*, **9**, 446.
Jolles, B.—(1962) *Br. J. Cancer*, **16**, 209.
Kaslaris, E. and Jull, J. W.—(1962) *Br. J. Cancer*, **16**, 479.
Klavins, J. V. and Kaufman, N.—(1962) *Acta cytol.*, **6**, 267.
Korenchevsky, V., Paris, S. K. and Benjamin, B.—(1950) *J. Geront.*, **5**, 120.
Krieg, A. F. and Reagan, J. W.—(1961) *Lab. Invest.*, **10**, 581.
Laffargue, P., Samso, A., Luscan, R. and Francois, H.—(1965). *Annls Anat. path.*, **8**, 85.
Lipschutz, A.—(1950) ' Steroid Hormones and Tumors '. Baltimore, Md (Williams and Wilkins).
Meisels, A.—(1964) *Acta cytol.*, **8**, 274.
Mueenuddin, G. and Zaman, H.—(1967) *Acta cytol.*, **11**, 205.
Murphy, E. D.—(1961) *J. natn. Cancer Inst.*, **27**, 611.
Nicol, T., Vernon-Roberts, B. and Quantock, D. C.—(1965) *J. Endocr.*, **33**, 365.
Pan, S. C. and Gardner, W. U.—(1948) *Cancer Res.*, **8**, 337.
Sedlis, A. and Stone, D. F.—(1965) *Acta cytol.*, **9**, 386.
Taki, I.—(1967) ' Uterine Carcinogenesis and Hormonal Imbalance '. *Jap. obstet. Gynaec. Soc.*, Monograph.
Ulfelder, H.—(1962) *Bull. Acad. Med. New Jersey*, **8**, 201.
Russfield, A. B.—(1966) ' Tumors of endocrine glands and secondary sex organs '. Public Health Service Publication No. 1332, Washington (U.S. Gov. Print. Office).